# Fusion rate and complications of oblique lumbar interbody fusion and transforaminal lumbar interbody fusion in the treatment of lumbar degenerative diseases: a meta-analysis

**DOI:** 10.3389/fsurg.2024.1374134

**Published:** 2024-04-30

**Authors:** Xun Xiao, Heng Duan, Xin Pan, Hua Zhao

**Affiliations:** Department of Orthopedics, Qilu Hospital of Shandong University, Cheeloo College of Medicine, Shandong University, Jinan, China

**Keywords:** lumbar degenerative disease, TLIF, OLIF, meta-analysis, fusion rate, complication rate

## Abstract

**Background:**

There currently exists some controversy about the efficacy of oblique lumbar interbody fusion (OLIF) and transforaminal lumbar interbody fusion (TLIF) in the treatment of lumbar degenerative diseases.

**Aim:**

This study compares the application effects of OLIF and TLIF in lumbar degenerative diseases by reviewing the literature and using meta-analysis.

**Methods:**

We included randomized controlled trials and cohort studies comparing TLIF and OLIF in the treatment of lumbar degenerative diseases. We searched for words such as “intervertebral disc degeneration,” “spinal fusion,” and “lumbar vertebrae” in the PubMed, Embase, and Cochrane Library databases. The search date was set from the establishment date of the database to October 2023. Two authors independently conducted document screening, data abstraction, and qualitative assessment. A meta-analysis was performed and adapted to RevMan5.3 software. The odds ratio (OR), weighted mean difference (WMD), and 95% CI were calculated by adopting a fixed-effect model (FEM) or a random-effect model (REM).

**Results:**

A total of 18 cohort studies were included with 1,550 patients, of whom 806 patients underwent TLIF (TLIF group) and 744 patients underwent OLIF (OLIF group). There were no significant differences found in the fusion rate [OR = 1.58 (0.95, 2.64), *P *= 0.08], complication rate [OR = 1.25 (0.93, 1.68), *P *= 0.14], and visual analog scale for back pain (VAS-BP) [WMD = 0.00 (−0.13, 0.14), *P *= 0.96] between the two groups. Compared with the TLIF group, the OLIF group had a lower Oswestry disability index (ODI) [WMD = −0.62 (−1.03, −0.20), *P *= 0.003], a higher foramen height (FH) [WMD = 2.03 (1.42, 2.46), *P *< 0.001], a higher disc height (DH) [WMD = 1.69 (1.17, 2.22), *P *< 0.001], and a shorter length of stay (LOS) [WMD = −1.80 (−2.55, −1.05), *P *< 0.001].

**Conclusion:**

In the treatment of lumbar degenerative diseases, compared with TLIF, OLIF has more advantages in terms of improving the lumbar function, restoring the FH and DH, and shortening the LOS. Both methods have comparable fusion rates, complication rates, and lumbar pain improvements. Due to the small amount of research and unclear assessment of the risk of bias, high-quality, large-sample randomized controlled studies are required to prove it.

## Introduction

Lumbar degenerative diseases, including lumbar degenerative spondylolisthesis, lumbar spinal stenosis, and degenerative scoliosis, involve the pathophysiological process of the natural aging and degeneration of the lumbar intervertebral disc and the paravertebral tissue (1). These diseases are the main cause of low back pain and leg pain in middle-aged and elderly people and can cause nerve injury in severe cases (2). With the aging of the global population, the incidence of lumbar degenerative diseases has been gradually increasing (3), placing a heavy burden on families and society. Conservative treatment is the first treatment of choice for lumbar degenerative diseases. If long-term conservative treatment is not effective, surgical treatment is needed. Lumbar interbody fusion is one of the most commonly used and effective methods of treating lumbar degenerative diseases. All kinds of surgical methods have their advantages and disadvantages. Therefore, the clinical treatment should be based on the patient's lesion characteristics, individual anatomical characteristics, and economic status. The surgical indications must be strictly grasped, and the best surgical method must be chosen to obtain the greatest effect. Transforaminal lumbar interbody fusion (TLIF) is widely used in clinical practice, while oblique lumbar interbody fusion (OLIF) is gradually becoming favorable as a new operation and has a good therapeutic effect. In recent years, an increasing number of studies have been conducted to compare the efficacies of TLIF and OLIF in the treatment of lumbar degenerative diseases, but there is still a lack of consistent conclusions. This study conducted a meta-analysis of this content to provide a reference for the selection of clinical surgical methods.

## Methods

### Eligibility criteria

The inclusion criteria are as follows: (1) study type: randomized controlled trials or cohort studies; (2) subjects: patients who are clinically diagnosed with lumbar degenerative diseases, including lumbar degenerative spondylolisthesis, lumbar spinal stenosis, and degenerative scoliosis, regardless of age, gender, and race; (3) intervention measures: surgical methods, namely, TLIF and OLIF, with study participants assigned to the TLIF group and the OLIF group, without limiting the surgical segment and fixation method; and (4) outcome indicators: fusion rate and complication rate. The literature included in the meta-analysis must include a main outcome indicator. The exclusion criteria are as follows: (1) non-clinical controlled study; (2) a combination of two or more types of surgery in the same treatment; (3) literature mainly in the form of personal experience, expert opinions, and animal experiments; (4) kinds of literature from which the full text cannot be obtained or the data cannot be extracted; (5) repetitively published literature; and (6) review, systematic review, and meta-analysis.

### Information sources

PubMed, Embase, and Cochrane Library databases were systematically retrieved. The search date was set from the establishment date of the database to October 2023. We collected research literature on TLIF and OLIF in the treatment of lumbar degenerative diseases and traced relevant references to supplement.

### Search strategy

In the abovementioned database, the system searches for words, such as “intervertebral disc degeneration,” “spinal fusion,” and “lumbar vertebrae,” adopts the search strategy of combining subject words with free words, and adjusts the search strategy according to the database. Taking PubMed as an example, the specific retrieval strategy was ((((“Intervertebral Disc Degeneration” [Mesh]) OR (((((((Intervertebral Disk Degeneration [Title/Abstract]) OR (Disk Degeneration [Title/Abstract])) OR (Disk Degradation [Title/Abstract])) OR (Degenerative Disc Disease [Title/Abstract])) OR (Disc Degradation [Title/Abstract])) OR (Degenerative Intervertebral Disc [Title/Abstract])) OR (Degenerative Intervertebral Disk [Title/Abstract]))) AND ((“Spinal Fusion” [Mesh]) OR (((((Spinal Fusions [Title/Abstract]) OR (Spondylodesis [Title/Abstract])) OR (Spondylodeses [Title/Abstract])) OR (Spondylosyndesis [Title/Abstract])) OR (Spondylosyndeses [Title/Abstract])))) AND ((“Lumbar Vertebrae” [Mesh]) OR (Vertebrae, Lumbar [Title/Abstract]))) AND ((((Transforaminal lumbar interbody fusion [Title/Abstract]) OR (TLIF [Title/Abstract])) OR (Oblique lumbar interbody fusion [Title/Abstract])) OR (OLIF [Title/Abstract])).

### Selection process

Two authors separately screened the literature. First, the title and the abstract of the literature were preliminarily browsed. After excluding unrelated literature, the full text of the abstract was further read according to the inclusion and exclusion criteria to determine whether it was included. After the screening completion, cross-checking was performed. Any objection must be resolved through a third-party consultation.

### Data collection process

Two authors independently extracted data according to a unified data extraction table. These included (1) common data: title, senior author, publication date, and research type; (2) research content: baseline data, intervention measures, grouping, outcome indicators, and outcome measurement data of the subjects; and (3) research characteristics: design scheme, inclusion and exclusion criteria, and measures to prevent bias. After the data extraction was completed, cross-checking was performed. Objections were resolved through a third-party consultation.

### Data items

The fusion rate and the complication rate were the primary outcome indicators. The visual analog scale for back pain (VAS-BP), Oswestry disability index (ODI), foramen height (FH), disc height (DH), and length of stay (LOS) were the secondary outcome indicators. This study mainly selected the last follow-up data for combined analysis.

### Study risk of bias assessment

Two authors independently used the Newcastle–Ottawa scale (NOS) to evaluate the quality of the included literature. The NOS was evaluated using three modules, namely, the selection (four items), comparability (one item), and exposure/outcome (three items) modules totaling to eight items. The score was based on the semiquantitative principle of the star system, with the highest score being two stars. The remaining items had the highest score of one star, which was equivalent to one score. The full score was nine scores, and a total score of ≥6 scores represents high-quality literature. After the quality evaluation was completed, cross-checking was performed. Objections were resolved through a third-party consultation.

### Effect measures

The weighted mean difference (WMD) and the odds ratio (OR) served as the effect analysis statistics for the quantitative data and qualitative data, respectively. The 95% confidence interval (95% CI) of each effect was provided.

### Synthesis methods

We used a fixed-effect model (FEM) or a random-effect model (REM) for the pooled data, and statistical heterogeneity between the aggregated data was evaluated by adopting the *I*^2^ statistics. If *P* > 0.1, *I*^2^ < 50% indicates that the heterogeneity is acceptable, and the FEM can be applied to the meta-analysis. If *P* < 0.1, *I*^2^ > 50% indicates a significant heterogeneity, and the REM can be applied to the meta-analysis after excluding the non-clinical sources of heterogeneity. The statistical analysis results of the combined effect size were represented by forest plots. All tests were two-tailed, and *P* < 0.05 was considered statistically significant.

### Reporting bias assessment

If there is heterogeneity, its source is first analyzed from both the methodological and clinical aspects. A subgroup analysis is then performed on factors that may lead to heterogeneity, such as study area, age, and internal fixation method, or a sensitivity analysis is performed. A funnel plot was used to check for publication bias. An asymmetric funnel plot suggested that there might be bias.

## Results

### Study selection

**Figure 1:**
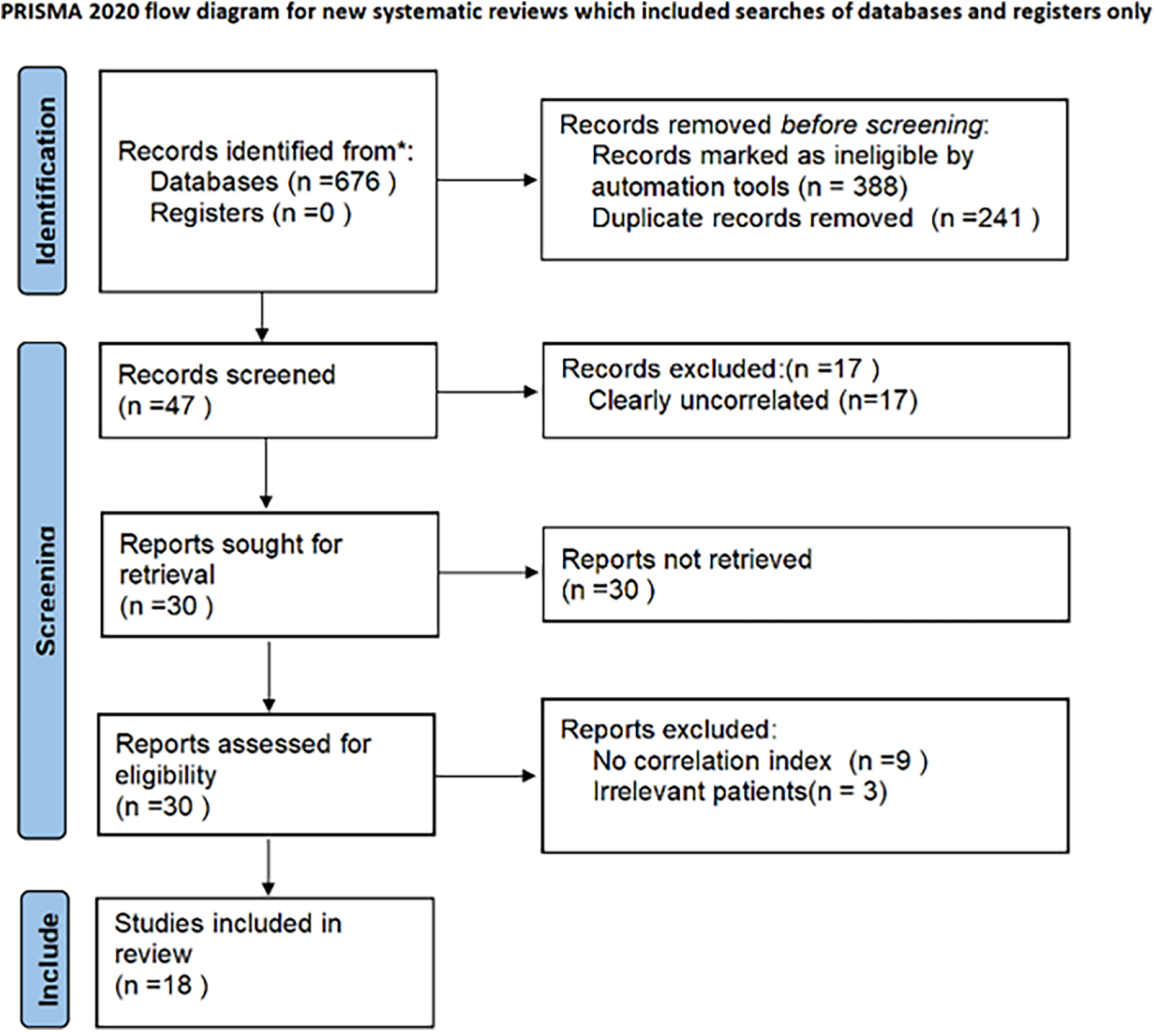
PRISMA flowchart.

A total of 676 articles were retrieved. Non-randomized controlled trials and non-observational studies were excluded using automated tools (*n* = 388), and duplicate studies were removed (*n* = 241). After the preliminary screening, 47 studies were obtained, and significantly unrelated studies were excluded (*n* = 17). After further obtaining the abstracts and full texts of 30 studies, 12 studies were excluded because 9 studies had no relevant indicators, and 3 studies did not meet the criteria for patient inclusion. Finally, 18 studies (4–21) were included in the meta-analysis. The screening process is displayed in Figure 1.

### Study characteristics

All selected studies were published between 2018 and 2023. Twelve studies were from China; three were from South Korea; two were from Japan; and one was from Thailand. The included studies were conducted comparatively to evaluate the efficacy of TLIF and OLIF in the treatment of lumbar degenerative diseases. The sample size of each group ranged from 25 to 79 cases. Most of the studies listed the age and gender of the subjects. Eleven studies used minimally invasive transforaminal lumbar interbody fusion (MIS-TLIF), and all studies used OLIF. The included studies described at least two outcome indicators and at most six outcome indicators. The characteristics of the included studies are summarized in Table 1.

**Table 1:** Basic information of the literature included in the study.

Study	Case		Age ($\bar{x}$, years)		Gender (male:female)		BMI ($\bar{x}$, kg/m^2^)		Operation type		Outcome
	T	O	T	O	T	O	T	O	T	O	
Lin et al. (4)	25	25	64.0	64.0	8:17	8:17	25.3	24.12	MIS-TLIF	OLIF	①②⑦
Mun et al. (5)	74	74	66.4	64.1	24:50	20:54	NA	NA	TLIF	OLIF	①③④⑤⑥
Sheng et al. (6)	55	38	60.62	65.29	25:30	8:30	NA	NA	MIS-TLIF	OLIF	②⑦
Du et al. (7)	37	28	52.8	53.6	23:14	16:12	22.5	22.8	TLIF	OLIF	①②
Han et al. (8)	33	28	53.6	50.4	15:18	12:16	24.1	24.9	MIS-TLIF	OLIF	①②③④⑥⑦
Li et al. (9)	35	28	59.3	57.5	8:27	7:21	NA	NA	TLIF	OLIF	②③④⑤⑥⑦
Kotani et al. (10)	38	33	64.7	63.1	25:13	15:18	23.2	22.7	MIS-TLIF	OLIF	①②
Takaoka et al. (11)	79	66	71.0	66	27:12	28:38	NA	NA	TLIF	OLIF	②③
Zhu et al. (12)	62	68	61.1	60.2	33:29	36:32	23.9	23.2	MIS-TLIF	OLIF	①②③④⑥⑦
Gao et al. (13)	60	53	59.23	58.42	28:32	23:30	24.67	23.74	MIS-TLIF	OLIF	②④⑦
Yingsakmongkol et al. (14)	30	30	67.1	63.0	6:24	8:22	NA	NA	MIS-TLIF	OLIF	①③④⑤⑦
Yoon et al. (15)	58	60	66.3	66.0	19:39	23:37	24.8	25.0	TLIF	OLIF	①②⑤⑦
Chen et al. (16)	30	28	61.13	63.00	17:13	15:13	25.23	25.89	MIS-TLIF	OLIF	②③④⑤⑥⑦
He et al. (17)	45	36	48.4	52.1	21:24	9:27	21.0	23.0	MIS-TLIF	OLIF	①③④⑤
Li et al. (18)	39	44	65.1	66.7	19:20	17:27	24.4	22.9	MIS-TLIF	OLIF	①②③④
Liu et al. (19)	35	35	62.86	64.40	14:21	14:21	24.79	24.81	MIS-TLIF	OLIF	②③④⑤⑦
Li et al. (20)	30	30	61.7	62.6	14:16	13:17	NA	NA	TLIF	OLIF	②③④
Wu et al. (21)	41	40	61.2	63.1	5:36	10:30	25.9	26.3	TLIF	OLIF	①②⑥⑦

T, TLIF group; O, OLIF group; BMI, body mass index; NA, missing value; ①, fusion rate; ②, complication rate; ③, visual analog scale for back pain; ④, Oswestry disability index; ⑤, foramen height; ⑥, disc height; and ⑦, length of stay.

### Risk of bias in the studies

The included studies were cohort studies, of which 17 were retrospective and 1 was prospective. The NOS scores of 16 studies were ≥6 points, which indicated a high research quality. The remaining two studies had an NOS score of only five points, which indicated that the research quality was moderate mainly because they did not correct for important confounding factors, such as age and gender, as shown in Table 2.

**Table 2:** Quality evaluation of the cohort study.

Study	Selection	Comparability	Exposure/outcome	Scores
Lin et al. (4)	⋆⋆⋆	⋆	⋆⋆	6
Mun et al. (5)	⋆⋆⋆	⋆	⋆⋆	6
Sheng et al. (6)	⋆⋆⋆	–	⋆⋆	5
Du et al. (7)	⋆⋆⋆	⋆	⋆⋆	6
Han et al. (8)	⋆⋆⋆	⋆	⋆⋆	6
Li et al. (9)	⋆⋆⋆	⋆	⋆⋆⋆	7
Kotani et al. (10)	⋆⋆⋆	⋆	⋆⋆	6
Takaoka et al. (11)	⋆⋆⋆	⋆	⋆⋆⋆	7
Zhu et al. (12)	⋆⋆⋆	–	⋆⋆	5
Gao et al. (13)	⋆⋆⋆	⋆	⋆⋆	6
Yingsakmongkol et al. (14)	⋆⋆⋆	⋆⋆	⋆⋆⋆	8
Yoon et al. (15)	⋆⋆⋆	⋆	⋆⋆	6
Chen et al. (16)	⋆⋆⋆	⋆	⋆⋆	6
He et al. (17)	⋆⋆⋆	–	⋆⋆⋆	6
Li et al. (18)	⋆⋆⋆	⋆	⋆⋆	6
Liu et al. (19)	⋆⋆⋆	⋆	⋆⋆	6
Li et al. (20)	⋆⋆⋆	⋆	⋆⋆⋆	7
Wu et al. (21)	⋆⋆⋆	⋆	⋆⋆⋆	7

⋆ stands for one score.

### Results of the syntheses

**Figure 2:**
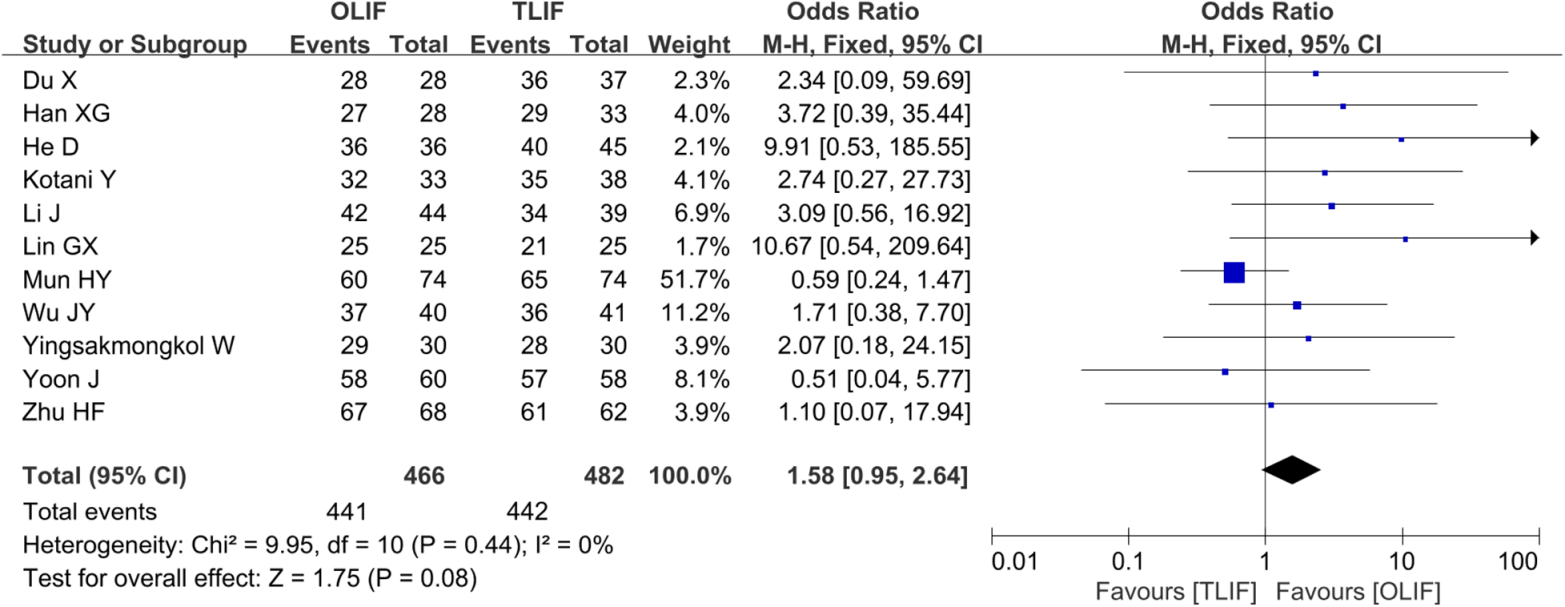
Forest plot for the comparison of the fusion rates.

Fusion rates were reported in 11 studies (4, 5, 7, 8, 10, 12, 14, 15, 17, 18, 21), and a total of 948 patients, of whom 466 belonged to the OLIF group and 482 belonged to the TLIF group. The heterogeneity between the studies was not significant (*I*^2 ^= 0%, *P *= 0.44); thus, the FEM was used for the combined analysis. The results showed that the fusion rate of the two groups was similar [OR = 1.58 (0.95, 2.64), *P *= 0.08], as shown in Figure 2.

**Figure 3:**
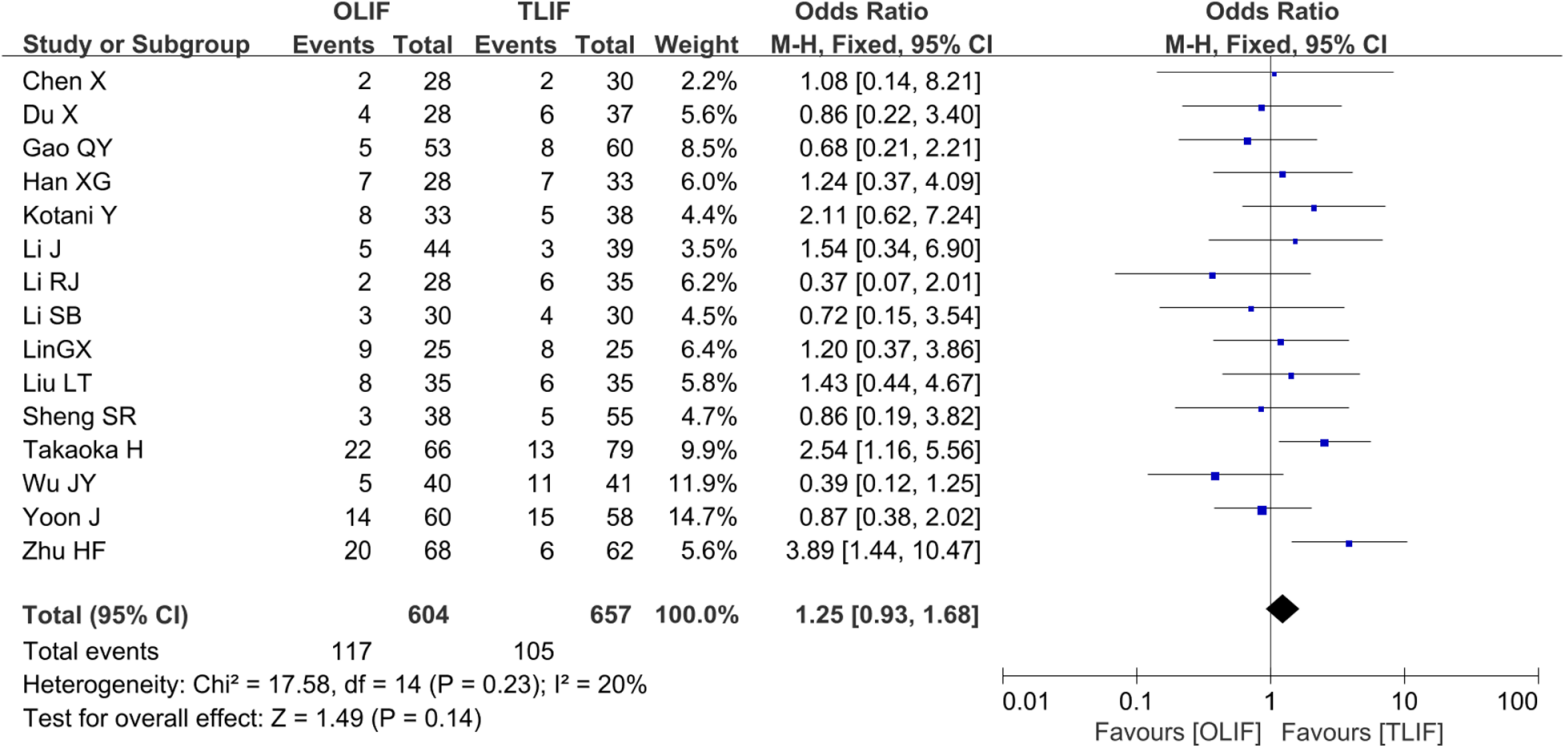
Forest plot for the comparison of the complication rates.

Complication rates were reported in 15 studies (4, 6–13, 15, 16, 18–21), and a total of 1,261 patients, of whom 604 belonged to the OLIF group and 657 belonged to the TLIF group. The heterogeneity between the studies was not significant (*I*^2 ^= 20%, *P *= 0.23); therefore, the FEM was used for the combined analysis. The results showed no significant difference in the complication rates between the two groups [OR = 1.25 (0.93, 1.68), *P *= 0.14], as shown in Figure 3.

**Figure 4:**
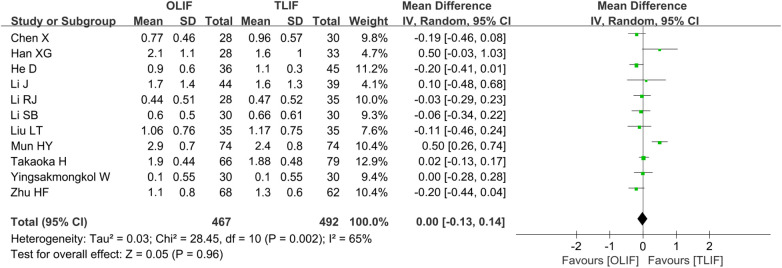
Forest plot for comparison of the VAS-BP scores. VAS-BP, visual analog scale for back pain.

VAS-BP scores were reported in 11 studies (5, 8, 9, 11, 12, 14, 16–20), and a total of 959 patients, of whom 467 belonged to the OLIF group and 492 belonged to the TLIF group. The heterogeneity among the studies was significant (*I*^2 ^= 65%, *P *= 0.002). After excluding the study of Mun, *I*^2^ decreased from 65 to 8%. However, the source of heterogeneity was not found; hence, the REM was used for the combined analysis. The results showed that the postoperative VAS-BP scores of the two groups were comparable [WMD = 0.00 (−0.13, 0.14), *P *= 0.96], as shown in Figure 4. After the combined studies were eliminated one by one by the sensitivity analysis, the *P*-values were all in the same direction, suggesting stable results.

**Figure 5:**
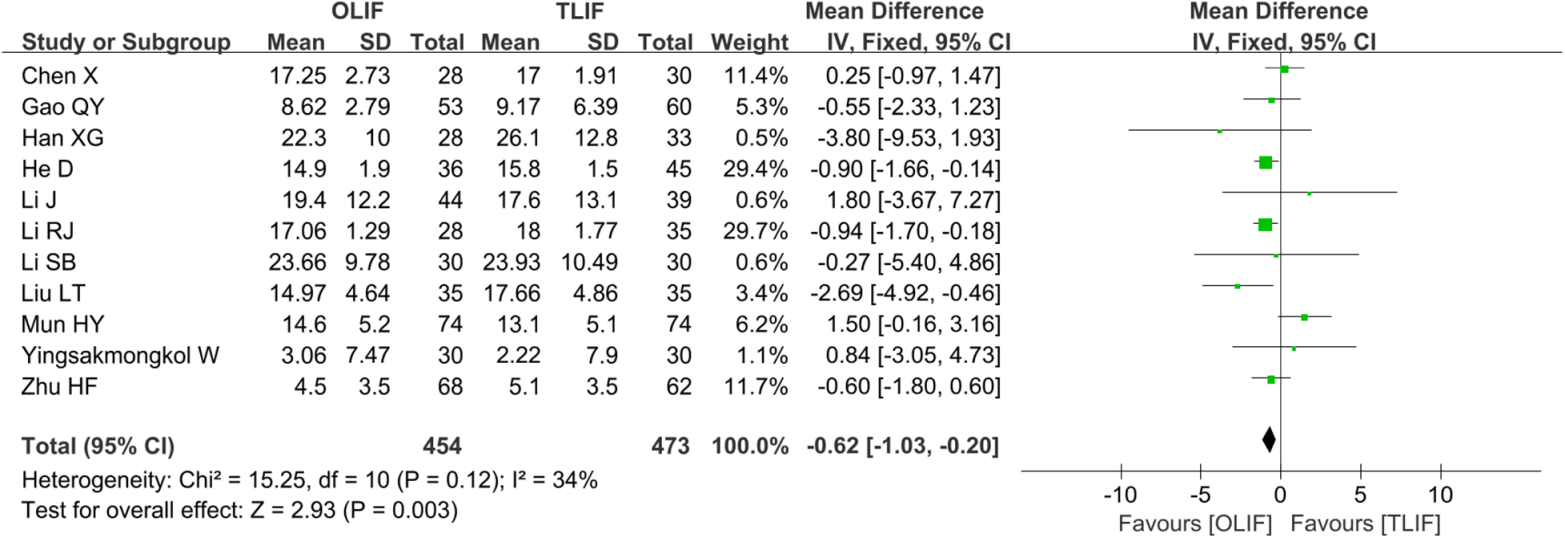
Forest plot for the comparison of the ODI. ODI, Oswestry disability index.

The 11 studies (5, 8, 9, 12–14, 16–20) that reported the ODI included 927 patients, of whom 454 belonged to the OLIF group and 473 belonged to the TLIF group. There was no significant heterogeneity among the studies (*I*^2 ^= 34%, *P *= 0.12); thus, the FEM was used for the pooled analysis. The results showed that the ODI in the OLIF group was lower than that in the TLIF group [WMD = −0.62 (−1.03, −0.20), *P *= 0.003], as shown in Figure 5.

**Figure 6:**
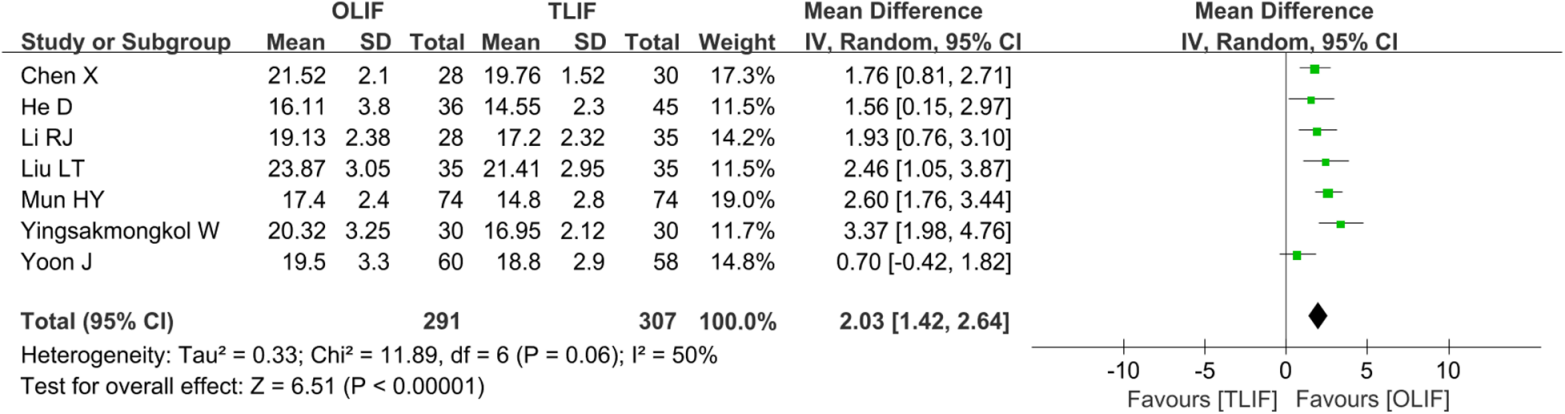
Forest plot for the comparison of the FH. FH, foramen height.

A total of seven studies (5, 9, 14–17, 19) reported FH and included 598 patients, of whom 291 belonged to the OLIF group and 307 belonged to the TLIF group. The heterogeneity between the studies was significantly smaller (*I*^2 ^= 50%, *P *= 0.06). After excluding the study of Yoon, *I*^2^ decreased from 50% to 9%. However, the source of the heterogeneity was not found; thus, the REM was used for the merger analysis. The results showed that the intervertebral FH in the OLIF group was higher than that in the TLIF group [WMD = 2.03 (1.42, 2.46), *P *< 0.001], as shown in Figure 6. After the combined studies were eliminated one by one by the sensitivity analysis, the *P*-values were all in the same direction, suggesting stable results.

**Figure 7:**
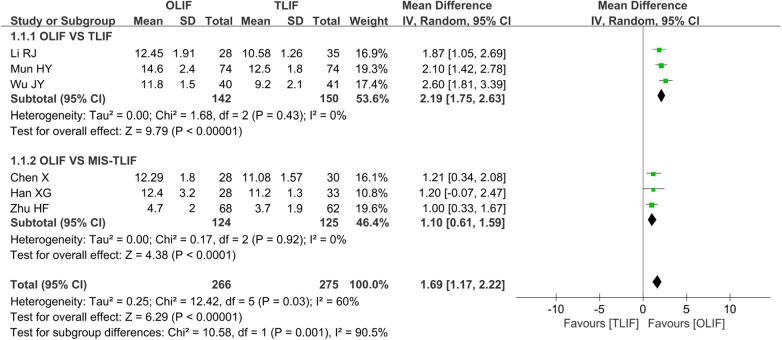
Forest plot for the comparison of the DH. DH, disc height.

A total of six studies (5, 8, 9, 12, 16, 21) reported DH and included 541 patients, of whom 266 belonged to the OLIF group and 275 belonged to the TLIF group. There was significant heterogeneity among the studies (*I*^2 ^= 60%, *P *= 0.03), and further analysis showed that the surgery type was the influencing factor of heterogeneity. The subgroup analysis based on the surgery type and the pooled analysis using a REM showed that the DH after OLIF was greater than that after TLIF [WMD = 2.19 (1.75, 2.63), *P *< 0.001] and MIS-TLIF [WMD = 1.10 (0.61, 1.59), *P *< 0.001], as shown in Figure 7.

**Figure 8:**
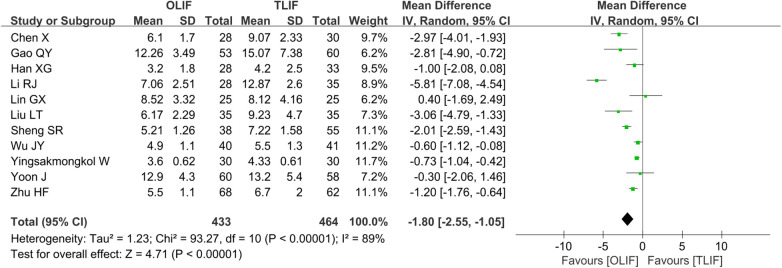
Forest plot for the comparison of the LOS. LOS, length of stay.

A total of 11 studies (4, 6, 8, 9, 12–16, 19, 21) reported LOS and included 897 patients, of whom 433 belonged to the OLIF group and 464 belonged to the TLIF group. The heterogeneity between the studies was significant (*I*^2 ^= 89%, *P *< 0.001). The one-by-one elimination method found that the *I*^2^ value did not change much, and the *P*-values all changed in the same direction, suggesting relatively stable results. We considered that the source of heterogeneity may be the difference in the level of surgeons, different internal fixation methods, postoperative care programs, etc.; hence, the REM was used for the merger analysis. The results showed that the LOS of the OLIF group was shorter than that of the TLIF group [WMD = −1.80 (−2.55, −1.05), *P *< 0.001], as shown in Figure 8.

### Reporting bias assessment

**Figure 9:**
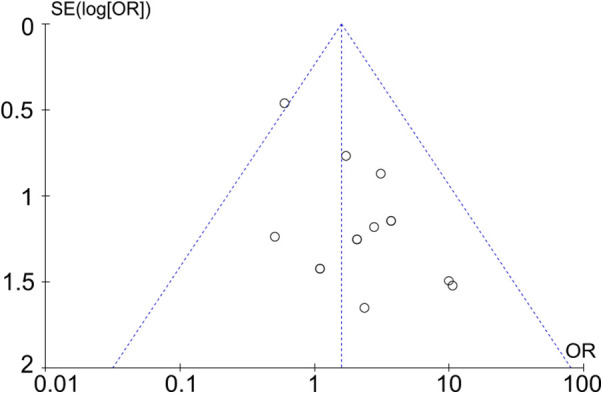
Funnel plot based on the fusion rate.

**Figure 10:**
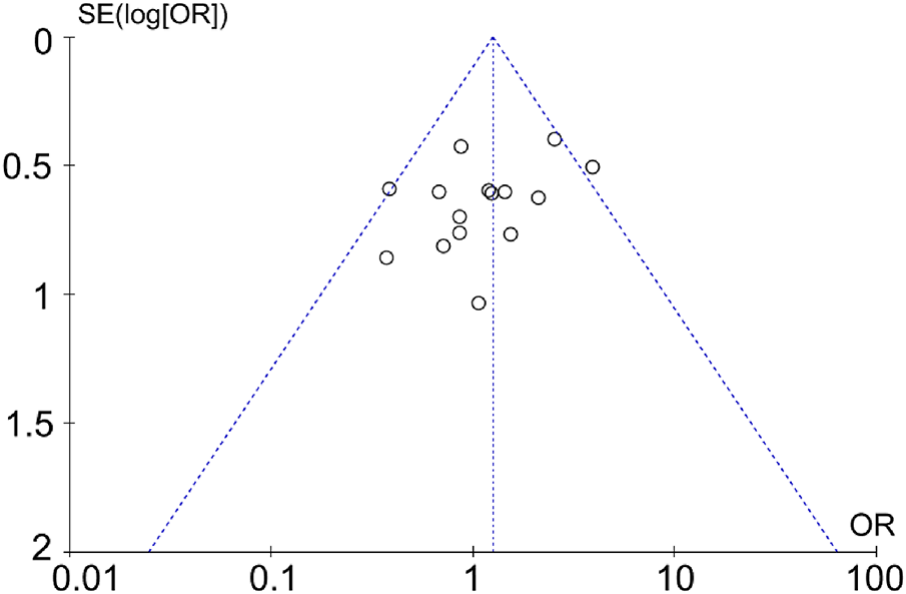
Funnel plot based on the complication rate.

The funnel plot was drawn according to the fusion rate and complication rate (Figures 9 and 10). There was literature outside the 95% confidence interval, and the left and right sides were asymmetric. Considering that there were many retrospective cohort studies included, there may be unpublished negative results, and there was a certain publication bias.

## Discussion

TLIF is another classic posterior fusion procedure developed based on posterior lumbar interbody fusion (PLIF), which is suitable for most lumbar degenerative diseases. It comprises the processes of spinal canal decompression and interbody fusion through the foramina, which effectively avoids excessive disturbance to the spinal dura and the nerve root during the operation and reduces the incidence of complications, such as nerve injury and cerebrospinal fluid leakage. Generally, this operation enters through the unilateral foramina, which can preserve the spinous process, the contralateral lamina, and the articular process and reduce the destruction of the lumbar posterior column (22). Although TLIF surgery, when compared to PLIF surgery, reduces the iatrogenic damage to the posterior column of the spine, it still causes damage to spine stability. The exposure and treatment of the intervertebral disc during surgery are limited to one side, and there is still a risk of damage to the dural sac and nerve root (23).

OLIF mainly accesses the working channel in front of the vertebra through the gap between the psoas major muscle and the abdominal aorta. This approach does not destroy the posterior column structure, minimizes damage to the muscle, and provides a large operating space. It enables a safer and more convenient cleaning of the intervertebral disc and allows for the placement of a larger area of the intervertebral fusion cage to improve the fusion rate (24). However, OLIF also has the risk of damaging blood vessels and sympathetic nerves, and its efficacy in cases of severe lumbar spondylolisthesis, severe spinal stenosis or bone stenosis, and large or prolapsed intervertebral disc herniation is not exactly defined (25).

Since the introduction of TLIF and OLIF in 1982 and 2012, respectively, they have become common lumbar fusion methods for the treatment of lumbar degenerative diseases and have yielded good surgical results. A meta-analysis showed that in treating lumbar degenerative diseases, OLIF was superior to TLIF in terms of intraoperative blood loss, LOS, ODI, DH, and FH. However, there was no significant difference in terms of the operation time, fusion rate, complications, VAS-BP, and sagittal imaging indices (26). Another meta-analysis showed that OLIF had advantages over TLIF in terms of the operation time, VAS-BP, DH, FH, and fusion rate, while the incidences of complications in the two groups were comparable (27).

This meta-analysis showed no significant difference in the fusion rate and the complication rate between the TLIF group and the OLIF group. The interbody fusion cage implanted in OLIF was generally larger than that in TLIF; thus, it had a larger contact area. In theory, the fusion rate of OLIF was higher. In an actual clinical process, TLIF can achieve a similar fusion rate because of the fixation effect of the posterior screw–rod system, the load of the cage is shared, and the posterior screw–rod provides a stable environment for interbody fusion (28). However, the fusion rate will also be affected by many factors, including the material of the fusion cage, placement position, whether or not to wear brace protection after surgery, and bone condition. In terms of complications, the TLIF group had more dural tears, cerebrospinal fluid leakage, and nerve root injury, while the OLIF group had more leg pain and numbness, sympathetic nerve injury, and hip flexion weakness. Most of the abovementioned conditions can be restored after conservative treatment and postoperative rehabilitation exercises.

In terms of restoring the FH and DH, the OLIF group had more advantages maybe because it allows the full expansion of the intervertebral space through the tension band of the posterior longitudinal ligament and implanting of a large cage with a certain inclination angle into the intervertebral space, which effectively disperses the load stress of the anterior column endplate and has better mechanical stability. TLIF can only implant a small fusion cage through a narrow operating space. It also removes one side of the lamina and facet joint while retaining the opposite side, such that the intervertebral space may not be effectively extended (29).

There was no significant difference in the VAS-BP scores between the two groups in terms of postoperative pain improvement. The postoperative ODI of the OLIT group was smaller than that of the TLIF group, indicating that OLIF had more advantages in improving postoperative lumbar function. TLIF directly accesses the spinal canal from the rear, expanding the spinal canal area and relieving nerve compression. By comparison, OLIF accesses from the oblique lateral space. Through the placement of a larger fusion cage, the height of the intervertebral space is expanded, the volume of the spinal canal is expanded, and indirect decompression is performed. Studies have shown that both direct decompression and indirect decompression can relieve nerve compression and pain (30). However, the difference is that OLIF minimizes tissue damage and postoperative trauma response, which are beneficial for improving lumbar function. The LOS of the OLIF group was shorter than that of the TLIF group. Our analysis was mainly related to the small invasiveness of OLIF, which was more conducive to postoperative recovery.

This meta-analysis had several limitations. (1) Most of the included studies are retrospective studies, with few prospective studies and a high risk of bias, which may affect the study results of the study. (2) The differences in the operation skills of different doctors, nursing programs, and internal fixation methods lead to a large heterogeneity between studies, which may affect the results. (3) The data included in the study mainly come from hospitals in different parts of the country, and the differences in the follow-up time of each study have an inevitable impact on the results. (4) In this meta-analysis, a funnel plot was employed to assess the publication bias, and all included studies fell outside the 95% CI, indicating the presence of a certain publication bias. Therefore, caution should be exercised when interpreting the findings of this study. Furthermore, it is imperative to enhance the retrieval strategy by comprehensively searching multiple databases and collecting all eligible research articles to improve the accuracy and reliability of the analysis. Additionally, more high-quality randomized controlled trials are warranted to provide robust support for drawing more compelling conclusions in this meta-analysis.

In summary, in the treatment of lumbar degenerative diseases, OLIF, when compared with TLIF, has more advantages in improving lumbar function, restoring FH and DH, and shortening LOS. Both had comparable fusion rates, complication rates, and lumbar pain improvements.

## Data Availability

The datasets presented in this study can be found in online repositories. The names of the repository/repositories and accession numbers are as follows: PubMed, Embase, and Cochrane Library.
